# Applying trans-theoretical model for blood donation among Spanish adults: a cross-sectional study

**DOI:** 10.1186/s12889-019-8046-9

**Published:** 2019-12-23

**Authors:** Lamyae Sardi, Ali Idri, Juan Manuel Carrillo de Gea, Ángel Toval, José Luis Fernández-Alemán

**Affiliations:** 1 Software Project Management research team, ENSIAS, University Mohammed V, Rabat, Morocco; 2 Complex Systems Engineering, University Mohamed VI Polytechnic, Ben Guerir, Morocco; 3 Department of Informatics and Systems, University of Murcia, Murcia, Spain; 4 Department of Human Anatomy and Psychobiology, School of Medicine, University of Murcia, Murcia, Spain; 5 Institute of Biomedical Research of Murcia (IMIB), Virgen de la Arrixaca University Hospital, University of Murcia, Murcia, Spain

**Keywords:** Blood donation, Trans-theoretical model, Behaviour change, Spanish adults, Cross-sectional study

## Abstract

**Background:**

Relying solely on altruistic appeals may fail to fulfil the increasing demand for blood supplies. Current research has largely been attempted to determine and understand motives that serve as blood donation drivers. The Trans-Theoretical Model of behaviour change (TTM) can be used to conceptualise the process of intentional blood donation behaviour.

**Methods:**

A cross sectional survey of Spanish adults was conducted. The final sample consisted of 504 individuals who were administered a self-report questionnaire including the measures of demographic characteristics, Stages of Change, Processes of Change, Self-efficacy and Decisional Balance. Data were analysed by frequency analysis, MANOVA/ANOVA and correlation analysis.

**Results:**

Findings indicated that most of the behavioural and cognitive processes of change, self-efficacy and physical cons differentiated participants across the stages of change of blood donation. In contrast, eligibility cons and pros were less influential in stage transitions. Furthermore, significant correlations were observed between TTM constructs except for the physical cons and the processes of change.

**Conclusions:**

The present study extensively supports and replicates the applicability of the TTM to blood donation behaviour change and offers important implications for the development of effective stage-matched interventions to increase blood donation.

## Background

In recent decades, researchers have identified a range of sociodemographic, organizational, psychological and physiological factors that impact the individual’s willingness to donate blood [1–3]. Although blood donation (BD) is considered as a purely prosocial behaviour, altruism and empathy were portrayed among the less significant motivations driving the BD decision [4]. Different theories and models of behaviour change have been applied to health contexts to assist in the design of behaviour change interventions. In this respect, six main theoretical perspectives to boost adherence to health behaviours have been identified (biomedical, behavioural, communication, cognitive, self-regulatory and stage perspectives) encompassing, each of them, different theories [5]. The most recurrently used theories are those within cognitive and stage perspectives [5]. The cognitive perspective includes theories that consider attitudes and beliefs as the locus of the individual’s behaviour. Of those theories, the Theory of Planned Behaviour (TPB) is the most widely cited and applied theory in predicting BD behaviour and intentions [6]. On the other hand, stage-based theories contend that individuals go through distinct stages as they learn and develop. The Trans-Theoretical Model (TTM) is the most prominent and widely applied among stage models [7].

### Theory of planned behaviour (TPB)

This theory evolved from the Theory of Reasoned Action (TRA) [8] which assumes that the intention to perform a particular behaviour acts as the best determinant and the most consistent predictor of that behaviour [9]. Intention, in turn, is believed to be directly affected by attitude that includes the individual’s positive or negative evaluations of the behaviour and subjective norms which reflect the individual’s perception of the social pressure exerted on him for the performance of the behaviour [8, 10].

Recognizing that the TRA omits the fact that behaviour may not always be function of voluntary control, Azjen [11] extended the theory to include the variable of behavioural control which reflects the ease or difficulty perceived in performing the behaviour. Conceptually, the perceived behavioural control is closely related to the notion of self-efficacy [12] since they are both concerned with the perceived ability to perform a behaviour [13]. Although the TPB has proved to be the most promising theory in predicting future blood donor behaviour [6], its predictive utility was generally improved by considering the incorporation of other constructs [14]. The extensions to the TPB include moral norm, anticipated regret, identity, self-categorization [15] donation anxiety and past behaviour, to cite but a few [16].

Nevertheless, many limitations have been levied against the use of the TPB in the prediction of blood donation intention and behaviour. It has been shown that these theories tend to focus on single, discrete acts rather than on repeated acts [16]. Moreover, models of attitude structure as the TPB, appear to offer very little in the way of selecting the highly significant predictors to guide interventions’ set up [6]. As a viable alternative to the TPB, researchers have begun to apply the TTM to conceptualize blood donation behaviour [17].

### The trans-theoretical model (TTM)

Originally, the TTM was developed to study nicotine addiction, it assesses the individuals’ readiness to quit smoking and provide them with well-established strategies to move towards smoking cessation [18]. More recently, the TTM has been applied in distinct cultures and ethnicities [19, 20] over numerous health behaviours [21] including exercise, dietary fat reduction, diabetes prevention, organ donation, etc.

The TTM consists of two major components: Stages of Change and Processes of Change [6]. The temporal dimension of this model is construed by these five exclusive stages of change: Pre-contemplation, Contemplation, Preparation, Action and Maintenance [6]. Each of these delineates the actual readiness and willingness of individuals for change. For instance, Pre-contemplation is the stage in which people are not planning to take action in the foreseeable future because they are unaware of the reason to change. Whilst, individuals in maintenance stage are being more confident to maintain the desired behaviour and are less tempted to relapse.

Ten processes of change have been suggested to facilitate the transition from one stage to the next and were classified into two categories: experiential and behavioural [22]. The experiential processes are used primarily for the early stage transitions and include a) Consciousness Raising, b) Dramatic Relief, c) Environmental Reevaluation, d) Social Liberation and e) Self-Reevaluation. The five behavioural processes used primarily for the later stage transitions include f) Stimulus Control, g) Helping Relationships, h) Counter Conditioning, i) Reinforcement Management and j) Self-Liberation. Each process of change intervenes uniquely at one transition.

Further, the trans-theoretical model was expanded to include two additional core constructs: Self-efficacy and Decisional balance. The application of self-efficacy has been found to have numerous implications in predicting blood donor behaviour. It is expected to increase as people progress through the stages [23]. However, it is particularly relevant at transition through the later stages [24]. Decisional balance reflects the individuals’ relative importance of the cons and pros of changing a specific behaviour. Recent research suggested that the pros are likely to increase in the earlier stages (e.g. pre-contemplation to contemplation) whereas the progress from contemplation to action involved a significant decrease in cons [21]. As such, individuals in the later stages endorse more positive aspects of change and more negative aspects in earlier stages. Given that the behavioural change is a function of the increases and decreases of pros and cons, decisional balance is of practical significance in developing tailored interventions to predict and enhance blood donors’ behaviour change.

Specifically, this study aims at applying TTM to blood donation behaviour among a Spanish population. To this end, five research questions (RQ) are investigated:

1. How are the recruited participants distributed over the five stages of change?
2. How do the Processes of Change vary across stages of change?
3. How do and Decisional balance (Cons/Pros) differ across stages of change?
4. How does Self-efficacy vary/differ across stages of change?
5. How is the correlation between the Processes of changes and Decisional balance, and Processes of changes and Self-efficacy?

## Materials and method

### Study design and participants

This study is cross-sectional in design and consisted of the dissemination of a paper-based survey among a population to gather their data with regards blood donation behaviour. All the procedures employed in this study were approved by the Ethics Committee of the University of Murcia. The recruitment phase led to the recruitment of 602 participants from the University of Murcia and Reina Sofia Hospital in Murcia. A total of 158 first and second-year students from the Faculty of Nursing were approached during lectures and were asked to fill in a survey designed to develop and validate processes of change, self-efficacy and decisional balance measures. The remainder of the sample (*N* = 444) was recruited at Reina Sofia Hospital where hospital staff, patients and their companions were handed the same questionnaire to fill in after giving their written informed consent to take part in this study. The questionnaires were completed under the supervision of the researcher who resolved any doubts. All participants were assured of anonymity and confidentiality.

### Data collection instruments and procedures

The study was quantitative, with data gathered through self-administered questionnaires. These questionnaires were designed such that they help to measure the four constructs of the TTM. A review of the literature in TTM and blood donation was conducted and integrated into the development of the questionnaire’s items to derive salient beliefs about blood donation [2, 3, 5, 25]. An adaptation to measures developed for blood donation and other content areas in previous studies [15, 17, 26], [24, 27] was conducted to refine the questionnaire’s items. The resulting questionnaire was translated into Spanish and consisted of five categories:

- Demographic characteristics

Various socio-demographic characteristics were assessed including age, gender, marital status and education level. Participants were also asked about their blood type.

b) Stage of Change

**Fig. 1:**
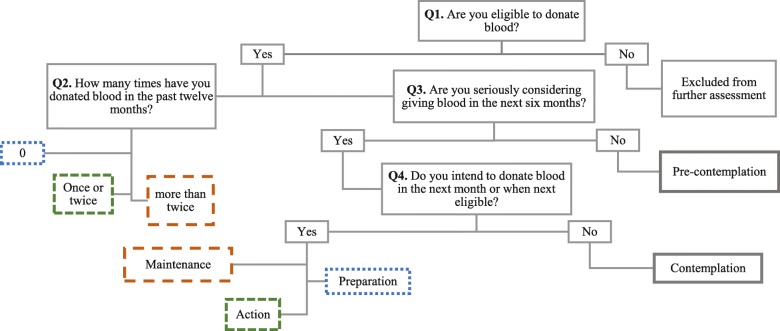
Algorithm of TTM Stages of Change for blood donation

Participants were asked to answer a short series of questions (Q1-Q4) regarding their past blood donation behaviour and their future intention to donate. Accordingly, a staging algorithm was developed and followed to place the participants in one of the exclusive five clusters for stage of change as shown in Fig. 1.

c) Processes of Change

A total of thirty items representing the ten Processes of Change (three items per process of change) were randomly comprised into the questionnaire. Participants were given a five-point scale ranging from “Never” to “Repeatedly” to rate the frequency in which they make use of a situation, thought and feeling to enhance their readiness to donate blood. Table 2 in the Appendix presents the description and the three proposed items for each process of change.

d) Self-efficacy

Self-efficacy is a measure of the extent to which an individual is confident in their ability to donate blood in the face of prospective hard situations (e.g. When I am feeling a physical discomfort). Eight items were designed to develop the self-efficacy scale. Responses were made on five-point scale, ranging from 1=” Not at all confident” to 5=” Extremely confident”. Table 3 in the Appendix show the eight statements used to measure self-efficacy.

e) Decisional Balance

Twelve items were designed to assess how an individual evaluates the pros and cons of blood donation. Six items were employed to reflect the Pros of blood donation (e.g. I will be helping to prevent blood shortages) and the remaining six items were evenly distributed among Physical Cons (e.g. I am likely to faint at the sight of blood) and Eligibility Cons (e.g. I might be told I am not eligible to donate blood). Participants responded on a five-level scale ranging from 1=” Not at all important” to 5=” Extremely important” to rate the importance of each item is in their decision to donate blood. Items used to measure the pros and cons of blood donation are depicted in Table 4 in Appendix.

### Statistical analysis

Frequency analysis was primarily performed to explore the distribution of the recruited individuals across Stages of Change with respect to blood donation behaviour. Multivariate and Univariate Analysis of Variance (MANOVA and ANOVA) tests along with post-hoc analyses were conducted to identify the differences in Process of Change subscales, Decisional Balance scales and Self-Efficacy scale with the five Stages of Change. For all TTM constructs, raw scores were converted to T-scores (Mean = 50, standard deviation [SD] = 10) in order to make comparisons easier in the magnitude of differences. In addition, Pearson correlations were examined between the different TTM variables. All statistical analyses applied in this study were performed using IBM SPSS 21.0.

## Results

### Sample

Of the 602 participants, 98 individuals were excluded from further assessment as they failed to answer (either by refusal or answering ‘I don’t know’) to the question concerning their eligibility to donate blood. The remainder sample (*N* = 504) was predominantly constituted by female (62.9%), and ages ranged from 18 to 80 with a mean of 27.32 (SD = 11.134). The reported education level showed that 46.8% of participants completed high school degree and 36.7% are currently enrolled or completed undergraduate degree program. Regarding blood types, A+ and O+ were the prevailing blood types among participants, accounting for 27.98 and 29.36% respectively. Table 5 in the Appendix depicts the frequency distribution of the recruited sample in relation to the demographic characteristics.

### Stages of change

All 504 participants were placed into four exclusive categories based on their responses to the aforementioned algorithmic staging questionnaire. The distribution by stage of change for the entire sample was as follows: Pre-contemplation 36.9% (*N* = 186), Contemplation 41.7% (*N* = 210), Preparation 9.3% (*N* = 47), Action 10.3% (*N* = 52) and Maintenance 1.8% (*N* = 9).

### Processes of change by stage of change

**Fig. 2:**
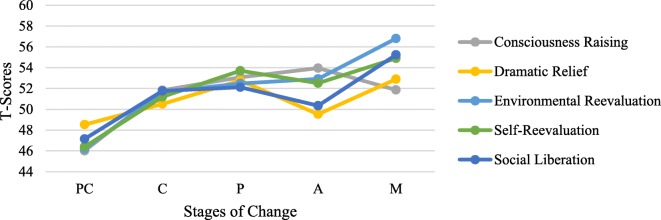
Behavioral Processes of Change across Stages of Change

**Fig. 3:**
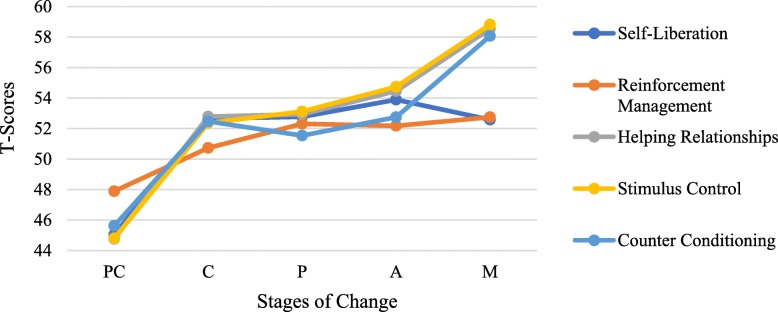
Experiential Processes of Change across Stages of Change

A one-way Multivariate Analysis of Variance (MANOVA) was conducted to test the hypothesis that there would be one or more mean differences between the ten Processes of Change and the five Stages of Change. A statistically significant MANOVA effect was obtained, Wilk’s Λ = .697, F (40,1814) = 4.54, *p* < .001, partial η2 = .086. These results revealed that the ten Processes of Change subscales were not equally and similarly triggered by Stage of Change. A series of one-way Analysis Variance (ANOVA) on each of the ten dependent variables was conducted as a follow-up tests to the MANOVA. Except for the experiential process of change ‘Dramatic Relief’, all effects were found to be statistically significant where the largest portion of variance was derived from Helping Relationships (η2 = .18), followed by Counter Conditioning (η2 = .17). The ANOVA values obtained were as follow: Consciousness Raising F(4,487) = 13.4, *p* < .001, η2 = .099; Dramatic Relief F (4,487) = 2.27, *p* = .061 ns, η2 = .018; Environmental Reevaluation F (4,487) = 11.62, *p* < .001, η2 = .09; Self-Reevaluation F (4,487) = 10.26, *p* < .001, η2 = .08; and Social Liberation F (4,487) = 20.52, *p* < .001, η2 = .05, Self-Liberation F(4,487) = 20.52, *p* < .01, η2 = .14; Reinforcement Management F(4,487) = 3.74, *p* < .05, η2 = .03; Helping Relationships F(4,487) = 25.46, *p* < .001, η2 = .18; Counter Conditioning F(4,487) = 24.46, *p* < .001, η2 = .17; and Stimulus Control F(4,487) = 16.08, *p* < .001, η2 = .12. Figures 2 and 3 show the experiential and behavioural Processes of Change comparison by Stage of Change, respectively.

### Decisional balance by stage of change

**Fig. 4:**
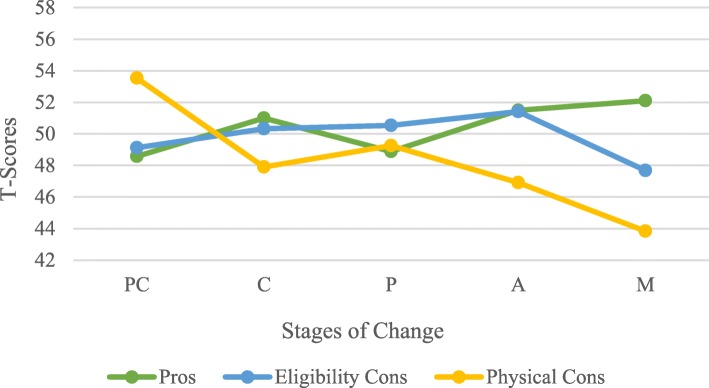
Decisional Balance scales across Stages of Change

MANOVA revealed that individuals in different stages of change varied significantly on the Decisional Balance scales (Pros, Eligibility and Physical Cons) of Blood Donation F (12,1272) = 5.819; *p* < .001; Wilks Λ = 0.868, partial η2 = .046. Follow-up ANOVA was conducted to compare the main effects of Decisional Balance scales across Stages of Change. All effects were found to be statistically non-significant at the .05 significance level except for Physical Cons. The main effect for Physical Cons yielded an F ratio of F (4,483) = 11.01; *p* < .001, the strength of the relationship, as indexed by η2 was equal to .084. For Eligibility Cons, the main effect yielded an F ratio of F (4,483) = .808; *p* = .52 ns; η2 = .007 while the Pros of blood donation obtained an F ratio of F (4,483) =1.966, *p* = .099 ns; η2 = .016. A Tukey HSD post-hoc tests further indicated that mean scores for the Pros and Eligibility Cons did not differ significantly across the five stages of change. The mean scores for Physical Cons were statistically significantly different between Pre-contemplation and Contemplation (*p* < .001), Pre-contemplation and Action (*p* < .001) and Pre-contemplation and Maintenance (*p* < .05) but not between Pre-contemplation and Preparation (*p* = .069). A graphical representation of T-scores on the decisional balance scales across the stages for blood donation is shown in Fig. 4.

### Self-efficacy by stage of change

**Fig. 5:**
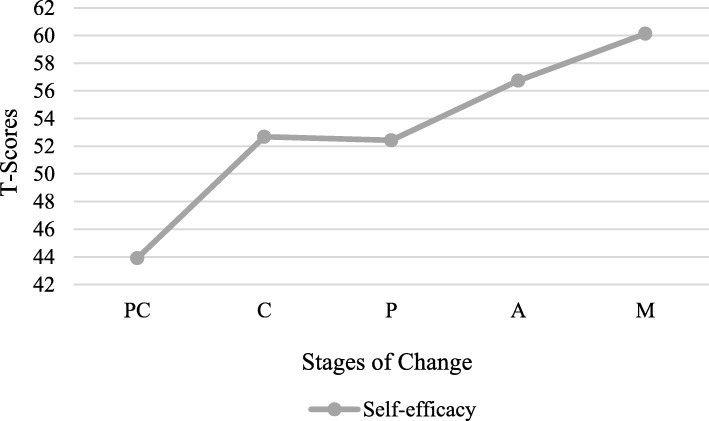
Self-Efficacy scale across Stages of Change

Self-efficacy scores yielded statistically significant differences across the five Stages of Change F (4.489) = 38.091 *p* < .001 with an effect size as indexed by η2 equal to .238. Post-hoc Tukey tests were performed to examine Self-efficacy mean comparisons across the five Stages of Change. The results revealed that Self-efficacy score were significantly higher in Action and Maintenance stages than in Pre-contemplation Stage. The variation of Self-Efficacy T-scores across stages is graphically shown in Fig. 5.

### Processes of change, decisional balance and self-efficacy

Table 1 illustrates the results of the correlation analysis to assess the relationships among the TTM constructs. Whilst all the correlation results were statistically significant, scores on both subscales of Processes of Change were not related to those on the Physical Cons. In addition, both behavioural and experiential Processes of Change for blood donation were positively correlated with Pros, Self-efficacy and Eligibility Cons. Physical Cons were positively related to Pros and Eligibility Cons. Overall, the strongest correlation yielded was that of Experiential Processes with Behavioural Processes (r = .793) followed by that of Eligibility and the benefits of blood donation (*r* = .525).

**Table 1:** Correlations between major TTM constructs

TTM constructs	EP	BP	Pros	EC	PC	SE
Experiential Processes (EP)	–	.793**	.389**.	.324**	.010	.379**
Behavioural Processes (BP)		–	.320**	.278**	−.036	.494**
Pros			–	.525**	.254**	.240**
Eligibility Cons (EC)				–	.272**	.261**
Physical Cons (PC)					–	−.193**
Self-Efficacy (SE)						–

*Note.* **. Correlation is significant at the 0.01 level (2-tailed)

## Discussion

The Stages of Change construct is one of the pillars of TTM theory. It reflects the individual’s motivational readiness to make a specific behaviour change. Accurate staging is perhaps the most crucial aspect of using TTM for developing health-related interventions [28]. To date, two major methods have been used for assigning stage classifications: staging algorithm and multidimensional questionnaire [29]. The staging algorithm approach uses a small number of questionnaire items to determine the participant’s stage. In the second approach, each Stage of Change is measured through a set of questionnaire items. With few exceptions (e.g. [17]), a number of applications of TTM to various health behaviours employed staging algorithms [19, 23, 30]. The staging algorithm used in this study to classify participants into one of the five stages of change depends on the assessment of recent past behaviour and the willingness to change behaviour. In fact, in order to be allocated to one of the earlier stages (Pre-contemplation, Contemplation, Preparation), participants are required to state their intent to donate blood in the near future. Nevertheless, Action and Maintenance stages require the demonstration of both intention and regular past experience of blood donation. Research suggests that past behaviour is a significant predictor of future behaviour for regular and experienced donors (5 or more previous donations), and intentions were predictive of occasional donors (4 or fewer previous donations) [31, 32]. For this reason, intentions outweigh past behaviour in the earlier stages of TTM. Consistent with previous research (e.g. [19, 23]), participants in this study were predominantly categorized into the pre-preparation stages accounting for 78.6% which mirrors the deterrence of participants in donating blood and the need to trigger a range of motives to induce progression across stages of change. This shall therefore contribute in the development and promotion of stage-matched interventions that harness the relevant and modifiable stage transitions determinants.

With regards to domination of the processes of change across the five stages of change, previous studies based on TTM demonstrated that experiential processes benefit progression through the earlier stages of behaviour change while behavioural processes have greater importance during later stages. In the current study, both sets of Processes of Change were significantly lower for individuals in Pre-contemplation than those in further stages. This result supports the hypothesis of the TTM, which is that the more advanced an individual is in Stage of Change, the more frequently they will use the Processes of Change. Indeed, pre-contemplators are unmotivated and unaware of the need to change, thus harnessing fewer strategies towards behaviour change [24, 33]. Moreover, individuals in Preparation stage were active on almost every process of change owing to the fact that prepared individuals are acutely motivated to experiment with changing behaviour employing therefore various methods that combine intention and behaviour criteria to improve their determination in favour of change. Aside from Consciousness raising and Self-liberation, all the processes of change peaked in the Maintenance stage. It is presumed that people in Maintenance stage do not apply Processes of Change as frequently as do people in earlier stages, yet, they need to stabilize their behaviour and work to avoid temptation and prevent relapses. In fact, most of the Processes of Change (e.g. counter conditioning, stimulus control) play a crucial role in helping individuals cope with high-risk situations that are associated to relapse. Based on ANOVA’s findings, Dramatic relief has no effect on the staging progress. Excepting Reinforcement Management, all the behavioural processes obtained very large effect sizes. Additionally, individuals in Action stage reported using Self-liberation strategy more often than those in other stages. This is not surprising, as people in Action stage need to learn how to consolidate their commitments, hence seeking interventions that strengthen their belief and increase their autonomy to change [34]. In accordance with previous studies, results demonstrated that people in Action and Maintenance stages emphasize the usage of both counter-conditioning and stimulus control for coping with temptations [29, 30]. Processes of Change offer theoretical valid strategies to help individuals progressively acquire new healthy behaviours. Given that specific Processes of Change are optimally effective at each stage of change, delivering tailored interventions that integrate the appropriate processes with the stages will promote behavioural change. However, failing to match processes of change to an individual’s stage of change can hamper the expected usefulness of interventions [35].

Alike processes of change, decisional balance also varies significantly across stages of change. The construct of decisional balance refers to the individual’s weighing the potential benefits and costs involved with changing behaviour. While most TTM studies put emphasis on two-dimensional scale to measure decisional balance [21], some TTM applications yielded a different scale of more than two factors [36, 37]. In the current study, the patterns of change in the pros and cons across the stages of change were found to be revealing. It was speculated that pros increase, and cons decrease from earlier to later Stages of Change defining a crossover pattern between Contemplation and Action stages. This result was achieved in Physical Cons and Pros and the crossover pattern occurs in the Preparation stage. However, the magnitude of change was not as large as expected. Physical cons of blood donation behaviour change significantly outweighed the pros in the Precontemplation stage and were lower than pros in the advanced stages. Indeed, individuals in earlier stages recall physical cons more often than those in further stages. In contrast, eligibility cons and pros did not yield a significant statistical difference across stages. Nonetheless, the pros increase slightly as individuals move toward the later stages with a small decrease in the Preparation stage. This result confirms that the progression across stages requires additional motivation by outbalancing the advantages of blood donation behaviour change over possible barriers. Moreover, eligibility cons did not decrease significantly across stages as did physical cons. This finding may have resulted from the possibility that regardless of their Stages of Change, blood donors may face rejection and deferral due to low haemoglobin level, high blood pressure and medication intake, to cite but a few [38]. Overall, Decisional Balance has demonstrated to be a good predictor through the stages of change.

Another major construct of TTM is Self-efficacy which refers to the perception and situational confidence that individuals have in their abilities to adopt and maintain the desired behaviour change even in difficult circumstances that often trigger relapse [12]. Research on TTM suggests that Self-efficacy increases in an almost linear fashion as the Stages of Change advances. Consistent with this, the Self-efficacy scores in the present study varied and rose significantly across stages. Contemplators had higher baseline of Self-efficacy scores than pre-contemplators and lower level than participants in advanced Stages of Change. In addition, participants in Preparation stage reported similar level of confidence to those in Contemplation stage. Perhaps individuals in preparation stage demonstrate ambivalence about their readiness to engage in the behavioural change. Moreover, participants identified in action and maintenance stages expressed the highest levels of self-efficacy with regard to blood donation in high-risk situations. This indicates that Self-efficacy is strongly influenced by performing the behaviour and that individuals in later stages are, by default, acting towards the behaviour change. The results obtained in this study provide evidence supportive of the applicability of the self-efficacy construct to actively change blood donation behaviour. This construct is genuinely considered a crucial resource to maintaining behaviour changes and preventing stage regression.

With respect to the associations among the TTM constructs, significant positive correlations were observed between the two dimensions of Processes of Change and Pros and Self-efficacy. Hence, TTM-based interventions that promote the usage of behavioural and cognitive Processes of Change should increase Pros and Self-efficacy accordingly. Eligibility cons were positively related to all TTM constructs and particularly to the pros of donating. A possible explanation is that even though individuals have eligibility concerns to donate blood, they outbalance their perception of the benefits of donating over these concerns. Moreover, scores on both cognitive and behavioural processes were not correlated with physical cons of blood donation. Consistent with this outcome, many studies have reported that physical concerns are less prominent in behaviour change [21, 39]. Additionally, a significant negative correlation was found between self-efficacy and physical cons. Therefore, it may be the case that as individuals gain confidence in their ability to donate blood, they start to attach little importance to the associated physical barriers. The highest correlation was found between the two dimensions of Processes of Change further supporting prior studies in which a tight association was perceived among processes [40].

### Study limitations

Despite the interest of this research, several limitations that had likely impacted the application or interpretation of the results of the present study are worth mentioning. First, due to the lack of a standardized measurement instrument for stage classification, the validity and reliability of staging algorithms have not yet been established [41]. To mitigate this constraint, the staging algorithm used in this study was elaborated on the basis of validated measures [19, 27]. Moreover, **t**he items developed to measure Processes of Change, Decisional Balance and Self-efficacy were derived from a selection of validated TTM measures in various health behaviours including blood donation. Despite the attempt to refine and adapt these measures to our study population, they may not have appropriately captured TTM constructs from the participants’ perception. Second, the questionnaire used in this study to gather data relied on a self-report format, leading to possible response bias due to a lack of validity and reliability [42]. Nevertheless, self-report measures are largely considered as a pertinent tool in health behavioural research [43, 44]. Finally, the size of the final sample was convenient, however, it comprises somewhat a restricted range of donors in advanced stages which was not representative of the rest of the blood donors’ population. This may jeopardize the generalizability of the findings of this study. It is, therefore, necessary to conduct further assessment that includes greater percentages of regular donors to benefit the yielded measures.

## Conclusion and future work

To the best of our knowledge, this is the first cross-sectional study to apply the Trans-Theoretical Model to blood donation behaviour within a Spanish population. The main purpose of the current study was to explore relationships among TTM constructs in a Spanish population with regards blood donation behaviour change. As behavioural and experiential Processes of Change, Self-efficacy and physical cons were found to be consistent variables of progression through stages of change for blood donation behaviour. However, concerns related to eligibility and perceived benefits of blood donation were influential in stage transitions. Overall, the results obtained are in general accordance with findings reported in previous studies and therefore, the applicability of TTM to blood donation behaviour is supported. More importantly, the current study offers important practical implications for the field of blood donation. The measures developed in this study can serve as a starting point for development of stage-matched interventions aimed at increasing blood donors’ intention. Accordingly, practitioners should learn behaviour change techniques related to the different constructs of TTM to build strategies that are suitable to the phases of the donations process.

Future work in this area should adopt a longitudinal perspective with a more evenly distributed sample of donors to explore in-depth how TTM construct help donors evolve across stages of change. Another potential future direction should focus on identifying strategies that harness Processes of Change, Decisional Balance and Self-efficacy for the development of TTM-grounded and appropriately tailored interventions targeted to blood donors. In this respect, persuasive strategies can play a paramount role in promoting behaviour change in these interventions. A further work may therefore aim at the implementation of a gamified blood donation app that integrates TTM constructs on the basis of this study. Gamification techniques have been found to positively affect health behaviours [45] and can be particularly harnessed in such a way to trigger the Processes of Change to stimulate stage transitions.

## Data Availability

The datasets generated during the current study are available from the corresponding author on request.

## Appendix

**Table 2:** Experiential and behavioural processes of change

	Processes of Change	Items
Experiential Processes	Consciousness Raising	
	Increase awareness and gain understanding about the behaviour change	I recall articles, posts and/or TV messages about donating blood.
		I look for information related to blood donation process.
		I seek out groups of people who can raise my awareness about how to become a blood donor.
	Dramatic Relief	
	Increase emotional experiences about the behaviour change	Portrayals of people whose lives are saved by blood donation affect me emotionally.
		I am moved by a person who helped save lives by donating blood.
		I get upset when I hear stories about people whose lives depend on regular blood transfusions.
	Environmental Reevaluation	
	Realize how the behaviour change affects physical and social environment	I am considering the idea that I could save lives by donating blood
		I stop to think about how donating blood would be beneficial for people in my community
		I realize that people who donate blood are a great source of inspiration to others.
	Self-Reevaluation	
	Assess how one feels with and without the behaviour change	I think that being a blood donor supports my view of myself as a caring and responsible person.
		I feel very competent and proud when I (decide to) donate blood.
		Being a non-donor makes me feel disappointed and helpless.
	Social Liberation	
	Harness environmental and social opportunities with the behaviour change	I am aware that society is actively encouraging and supporting people to become blood donors.
		I notice that there are more opportunities to donate blood in my community.
		I see more companies and organizations hosting and sponsoring blood drives.
Behavioural Processes	Self-Liberation	
	Choose and commit to act or believe in the ability to change	I make commitments to myself to donate blood.
		I recognize I have the energy needed to be a blood donor.
		I tell myself that I can be a blood donor despite the fact that my relatives and friends don’t support my decision.
	Reinforcement Management	
	Reward oneself or be awarded for making steps towards behaviour change	I can expect to be praised and appreciated by others for donating blood
		I feel respected in society for being a blood donor.
		I reward myself with a treat after donating blood.
	Helping Relationships	
	Trust and accept the support of others that encourage the desired behaviour	I share with someone my thoughts and feelings about blood donation.
		There are special people around me that encourage me and improve my willpower to continue donating blood.
		I have a friend on whom I can count to come with me when I want to donate blood.
	Counter Conditioning	
	Substitute healthy behaviours and thoughts for the problem behaviour	I keep in mind that blood donation is a simple and safe process to overcome the fear of donating.
		When I am hesitant to donate blood, I remind myself that it helps save lives.
		Whenever I feel tempted to reassess being a blood donor, I begin to think about all the health benefits it offers.
	Stimulus Control	
	Avoid cues for unhealthy habits and add stimuli that encourage alternative behaviours	I make sure I know when and where nearby blood drives are held.
		I schedule my blood appointments.
		I keep around any source of information associated with blood donation to reconsider my reasons for donating blood.

**Table 3:** Items for decisional balance scale

Decisional balance items	
Pros	I may help save someone’s life.
	Donating blood will reduce the risk of getting serious health conditions.
	I will get a free of cost health check-up.
	Donating blood will help me burn calories
	I will be helping to prevent blood shortages
	I will set a good example and inspiration for people around me.
Physical Cons	I am likely to faint at the sight of blood.
	Donating blood depletes the calcium levels in the body.
	Donating blood is an uncomfortable experience because I am afraid of needles.
Eligibility Cons	The blood bank might reject my blood due to low level of my Haemoglobin.
	I may find out I have a disease.
	I might be told I am not eligible to donate blood.

**Table 4:** Items for self-efficacy scale

Self-efficacy	
1) When I am very anxious and stressed.	
2) When I am feeling a physical discomfort.	
3) When I witness a bad blood donation experience (e.g. Someone fainting).	
4) When I realize I have not donated for a long while.	
5) During or after experiencing personal problems (e.g. family, financial).	
6) When I have other time commitments.	
7) When I remember having a negative reaction to donating that caused me light-headedness and nausea.	
8) After recovering from an illness or an injury	

**Table 5:** Demographic characteristics by stage of change

Characteristic	TTM Stages											
	Precontemplation *N* = 186		Contemplation *N* = 210		Preparation *N* = 47		Action *N* = 52		Maintenance *N* = 9		Total *N* = 504	
	*n*	%	*n*	%	*n*	%	*n*	%	*n*	%	*N*	%
Gender												
Female	101	54.3	137	65.24	35	74.47	38	73.08	6	66.67	317	62.9
Male	85	45.7	73	34.76	12	25.53	14	26.92	3	33.33	187	37.1
Age range												
< 21	43	23.12	81	38.57	22	46.81	20	38.46	5	55.56	171	33.92
21–30	76	40.86	82	39.05	11	23.4	23	44.23	3	33.33	195	38.7
31–40	18	9.68	20	9.52	4	8.51	4	7.69	0	0	46	9.12
41–50	26	13.98	13	6.19	8	17.02	1	1.92	0	0	48	9.52
51–60	15	8.06	1	0.48	0	0	1	1.92	0	0	28	5.56
> 60	2	1.08	0	0	0	0	0	0	0	0	2	0.4
Don’t know/ No answer	6	3.23	5	2.38	2	4.25	1	1.92	0	0	14	2.77
Education level												
Primary	8	4.3	7	3.33	2	4.25	0	0	0	0	17	3.37
Secondary	84	45.16	96	45.71	27	57.45	26	50	3	33.33	236	46.83
Undergraduate	57	30.64	91	43.33	14	29.79	18	34.61	5	55.56	185	36.71
Postgraduate	36	19.35	14	6.67	2	4.25	8	15.38	1	11.11	61	12.1
Don’t know/ No answer	1	0.54	2	0.95	2	4.25	0	0	0	0	5	0.99
Marital Status												
Single	129	69.35	175	83.33	36	76.6	46	88.46	7	77.78	393	77.98
Married	45	24.19	31	14.76	9	19.15	5	9.61	1	11.11	91	18.05
Divorced	8	4.3	2	0.95	1	2.13	1	1.92	0	0	12	2.38
Separated	3	1.61	1	0.48	1	2.13	0	0	1	11.11	6	1.19
Widowed	1	0.54	1	0.48	0	0	0	0	0	0	2	0.4
Blood type												
A-	8	4.3	9	4.28	0	0	1	1.92	0	0	18	3.57
A+	45	24.19	59	28.09	12	25.53	20	38.46	5	55.56	141	27.98
O+	50	26.88	66	31.43	14	29.79	17	32.69	1	11.11	148	29.36
O-	15	8.06	29	13.81	6	12.76	5	9.61	0	0	55	10.91
B-	4	2.15	2	0.95	0	0	1	1.92	0	0	7	1.38
B+	12	6.45	11	5.24	3	6.38	5	9.61	3	33.33	34	6.75
AB+	12	6.45	14	6.67	1	2.13	1	1.92	0	0	28	5.56
AB-	0	0	1	0.48	1	2.13	0	0	0	0	2	0.4
Don’t know/ No answer	40	21.5	19	9.05	10	21.28	2	3.85	0	0	71	14.09

## Abbreviations

ANOVA: Analysis of Variance
BD: Blood donation
MANOVA: Multivariate Analysis of Variance
RQ: Research Question
SD: Standard Deviation
TPB: Theory of Planned Behaviour
TRA: Theory of Reasoned Action
TTM: Trans-Theoretical Model
